# The impact of *DNMT3A* variant allele frequency and two different comutations on patients with de novo cytogenetically normal acute myeloid leukemia

**DOI:** 10.1002/cam4.5764

**Published:** 2023-03-13

**Authors:** Xian Chen, Chuchu Tian, Zhuanghui Hao, Lingang Pan, Minglin Hong, Wei Wei, Daniel Muteb Muyey, Hongwei Wang, Xiuhua Chen

**Affiliations:** ^1^ Institute of Hematology The Second Hospital of Shanxi Medical University Taiyuan China; ^2^ Department of Genetic Medicine Shanxi Medical University Jinzhong China

**Keywords:** acute myeloid leukemia, *DNMT3A* mutations, variant allele frequency

## Abstract

To refine the biological and prognostic significance of *DNMT3A* mutations in acute myeloid leukemia (AML), we assessed the impact of *DNMT3A* variant allele frequency (VAF) and its comutations in this study. Using targeted next‐generation sequencing, we analyzed 171 adult patients with de novo cytogenetically normal AML for *DNMT3A* mutations and associated comutations. *DNMT3A* ^mut^ was detected in 35 patients. *DNMT3A* ^mut^ patients were divided into *DNMT3A* ^High^ and *DNMT3A* ^Low^ using a cut‐off VAF value of 42%. We observed that *DNMT3A* ^High^ patients at diagnosis had increasing white blood cell (WBC) counts (*p* < 0.001) and a higher lactate dehydrogenase (LDH) level (*p* = 0.027), and were associated with lower complete remission (CR) rate (*p* = 0.015) and shorter overall survival (OS) (*p* = 0.032) than *DNMT3A* ^Low^ patients. We classified two different comutated genetypes, including *DNMT3A* ^mut^ *NPM1* ^mut^ *FLT3‐ITD* ^mut^ and *DNMT3A* ^mut^ *IDH1/IDH2* ^mut^. Patients with *DNMT3A* ^mut^ *NPM1* ^mut^ *FLT3‐ITD* ^mut^ showed worse OS (*p* = 0.026) and relapse‐free survival (RFS) (*p* = 0.003) than those with *DNMT3A* ^mut^ *IDH1/IDH2* ^mut^, and showed a shorter OS (*p* = 0.027) than those with *DNMT3A* ^wt^ *NPM1* ^mut^ *FLT3‐ITD* ^mut^. We also observed that patients with *DNMT3A* ^mut^ *IDH1/IDH2* ^mut^ had higher platelet counts (*p* = 0.009) and a lower BM blast percentage (*p* = 0.040) than those with *DNMT3A* ^wt^ *IDH1/IDH2* ^mut^. In multivariate analyses, *DNMT3A* ^High^ was independently associated with a lower CR rate (OR = 5.883; *p* = 0.004) and shorter OS (HR = 3.768; *p <* 0.001). *DNMT3A* ^mut^ *NPM1* ^mut^ *FLT3‐ITD* ^mut^ independently affected worse OS (HR = 6.030; *p <* 0.001) and RFS (HR = 8.939; *p <* 0.001). Our findings might be potentially useful for predicting clinical outcomes.

## Graphical abstract

Using targeted next‐generation sequencing, we analyzed 171 adult patients with de novo cytogenetically normal AML for *DNMT3A* mutations and associated comutations. *DNMT3A* ^mut^ was detected in 35 patients. *DNMT3A* ^mut^ patients were divided into *DNMT3A* ^High^ and *DNMT3A* ^Low^ using a cutoff VAF value of 42%. We observed that *DNMT3A* ^High^ patients at diagnosis had increasing WBC counts and a higher LDH level, and lower CR rate and shorter OS than *DNMT3A* ^Low^ patients. We classified two different comutations, including *DNMT3A* ^mut^ *NPM1* ^mut^ *FLT3‐ITD* ^mut^ and *DNMT3A* ^mut^ *IDH1/IDH2* ^mut^. Patients with *DNMT3A* ^mut^ *NPM1* ^mut^ *FLT3‐ITD* ^mut^ showed worse OS and RFS than those with *DNMT3A* ^mut^ *IDH1/IDH2* ^mut^.

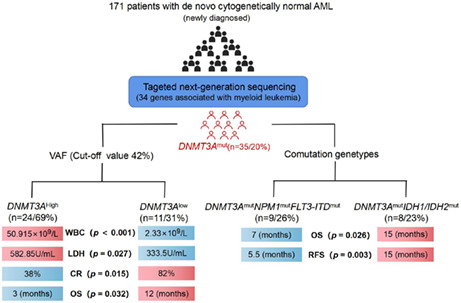

## 1 INTRODUCTION

*DNMT3A* belongs to a family of *DNMTs*, including *DNMT1*, *DNMT3A*, and *DNMT3B*, which encodes a DNA methyltransferase that is thought to regulate de novo DNA methylation modification, rather than maintenance methylation.^1^, ^2^ By regulating DNA methylation, *DNMT3A* may regulate the growth of hematopoietic stem cells (HSCs) into a predominantly granulocytic lineage during normal hematopoiesis.^3^, ^4^, ^5^ Studies in mice have demonstrated that deletion of *DNMT3A* could cause HSC persistent self‐renewal and inefficient differentiation.^6^, ^7^, ^8^

Somatic *DNMT3A* mutations are observed in various types of adult myeloid and lymphoid neoplasms. They are more frequently occurred (23%–37%) in adult patients with cytogenetically normal acute myeloid leukemia (CN‐AML),^9^, ^10^, ^11^, ^12^, ^13^, ^14^ but very rarely found in pediatric or adolescent blood cancers.^15^, ^16^, ^17^ *DNMT3A* mutations are usually heterozygous in AML.^9^, ^18^, ^19^, ^20^ In leukemogenesis, the mutant protein can dimerize with wild‐type DNMT3A, but homotetramers with more potent activity cannot be formed. The resulting low level of DNMT3A homotetramer results in significantly reduced methyltransferase activity and genome‐wide hypomethylation in patients.^21^, ^22^, ^23^, ^24^ In contrast to wild‐type DNMT3A, more recent research suggests that it could interact with EZH2, which is the catalytic component of Polycomb repressive complex 1, leading to the down‐regulation of genes associated with hematopoietic differentiation.^25^

Although DNMT3A mutations are more common in clonal hematopoiesis and appear to be relatively early events in leukemogenesis, the clinical effect of *DNMT3A* mutations on CN‐AML remains inconclusive. Previous studies vary regarding the impact of *DNMT3A* mutations. Some studies found a significantly worse overall or event‐free survival (EFS).^9^, ^20^, ^26^, ^27^, ^28^, ^29^, ^30^, ^31^, ^32^, ^33^ Still, others found no significant association with overall and EFS.^11^, ^18^, ^34^, ^35^, ^36^ These conflicting results may be due to the genetic heterogeneity of *DNMT3A‐*mutated CN‐AML. Hence, it is crucial to further refine the genetic subclassification for a better understanding of the clinical effect of *DNMT3A* mutations in adult CN‐AML.

We analyzed a cohort of 171 adult patients with de novo CN‐AML for *DNMT3A* mutations and associated comutations using targeted next‐generation sequencing (NGS). We further investigated the clinical impact of *DNMT3A* variant allele frequency (VAF) and two different comutated genetypes on these patients, which might help to further refine biological and prognostic implications of *DNMT3A* mutations in de novo CN‐AML.

## 2 METHODS

### 2.1 Patients group

We conducted a retrospective review of NGS analyses performed on clinical bone marrow samples from adult patients with De novo CN‐AML who presented to The Secondary Hospital of Shanxi Medical University Hematology Center in China between February 2017 and January 2021 in this study. We identified 171 newly diagnosed adult patients with de novo CN‐AML (90 males and 81 females; median age, 53 years; age range, 19–86 years). The clinical samples were unpaired design. All patients provided informed written consent. This study complied with the Helsinki declaration and was approved by the ethical board of the Second Hospital of Shanxi Medical University. The patient's data including age, sex, hematological parameters, blasts in bone marrow aspirates, and prior history of cytotoxic chemotherapy or radiotherapy were obtained from the medical records at diagnosis. De novo AML defined the patient as having no antecedent myeloid malignancy, cytotoxic therapy or radiotherapy before the diagnosis. A total of 168 patients were subjected to anti‐cancer therapies. Among these patients, 125 were treated with high‐intensity induction chemotherapy regimens and 43 received low‐intensity induction chemotherapy regimens. The dose and course of treatment were performed according to the Chinese Guidelines for the Diagnosis and Treatment of adult acute myeloid leukemia (non‐acute promyelocytic leukemia) (2017 edition). Twenty‐one patients without concomitant *DNMT3A* mutations but in the intermediate or adverse‐risk group underwent allogous‐ hematopoietic stem cell transplantation (allo‐HSCT) after complete remission (CR). The CR and recurrence were determined according to the ELN2017 recommendations. Overall survival (OS) was calculated as the period from the date of diagnosis to death or to the date of last observation. Relapse‐free survival (RFS) was calculated as the period from the first CR to relapse, death or the last observation.

### 2.2 Molecular analysis

A targeted NGS study was used to analyze the DNA of fresh bone marrow samples at initial diagnosis. Blood genomic DNA was isolated by Mini Blood DNA kit (Qiagen or OMEGA) and quantified with the NanoDrop spectrophotometer. We sequenced the mutation hotspots or entire coding regions of 34 genes associated with myeloid leukemia, which contains *FLT3*, *JAK2*, *KIT*, *MPL*, *CALR*, *CSF3R*, *PDGFRA*, *CEBPA*, *NRAS*, *KRAS*, *NPM1*, *TP53*, *RUNX1*, *GATA2*, *WT1*, *TET2*, *DNMT3A*, *IDH1*, *IDH2*, *ASXL1*, *BOCR*, *BOCRL1*, *CBL*, *ETV6*, *EZH2*, *MLL*, *NOTHCH2*, *PHF6*, *SF3B1*, *SRSF2*, *SH2B3*, *SETBP1*, *U2AF1*, *ZRSR2*. The regions analyzed included mutational hotspots or the coding sequence of 34 genes. In Brief, 50 ng genomic DNA was used for each reaction. DNA samples from all patients were sequenced and analyzed on a high‐throughput sequencing platform, the MiSeq next‐generation sequencing instrument (Illumina). VAF was observed with a specific DNA sequence variation matching divided by the percentage of the overall coverage of the site. VAF greater than 5% was considered to be the presence of a mutation. VAF cut‐off value was to use the optimal cutoff method with the Cutoff Finder web application (http://molpath.charite.de/cutoff). Data of patients with *DNMT3A* mutations were uploaded from a tab‐separated table. The VAF of *DNMT3A* mutations and OS survival variable were selected from the table. The optimal cut‐off point was determined by choosing “ROC curve (Manhattan distance)” method.

### 2.3 Statistical analysis

The clinical features of the patients were described using descriptive statistics. Differences between groups were analyzed by the chi‐squared or Fisher exact test for categorical variables and non‐parametric Mann–Whitney U test for continuous variables. Survival analysis using the Kaplan–Meier method and the log‐rank test, including the OS and RFS. Regarding the multivariate analysis of prognostic factors, a Cox‐proportional hazards regression model was used for survival endpoints and a logistic regression model was used for CR. Bilateral *p* < 0.05 prompt difference was statistically significant. All statistical procedures were performed using SPSS software package version 25.0 and Graphpad Prism™ 8.30.

## 3 RESULTS

### 3.1 DNMT3A mutations characterization in adult patients with de novo CN‐AML

**FIGURE 1:**
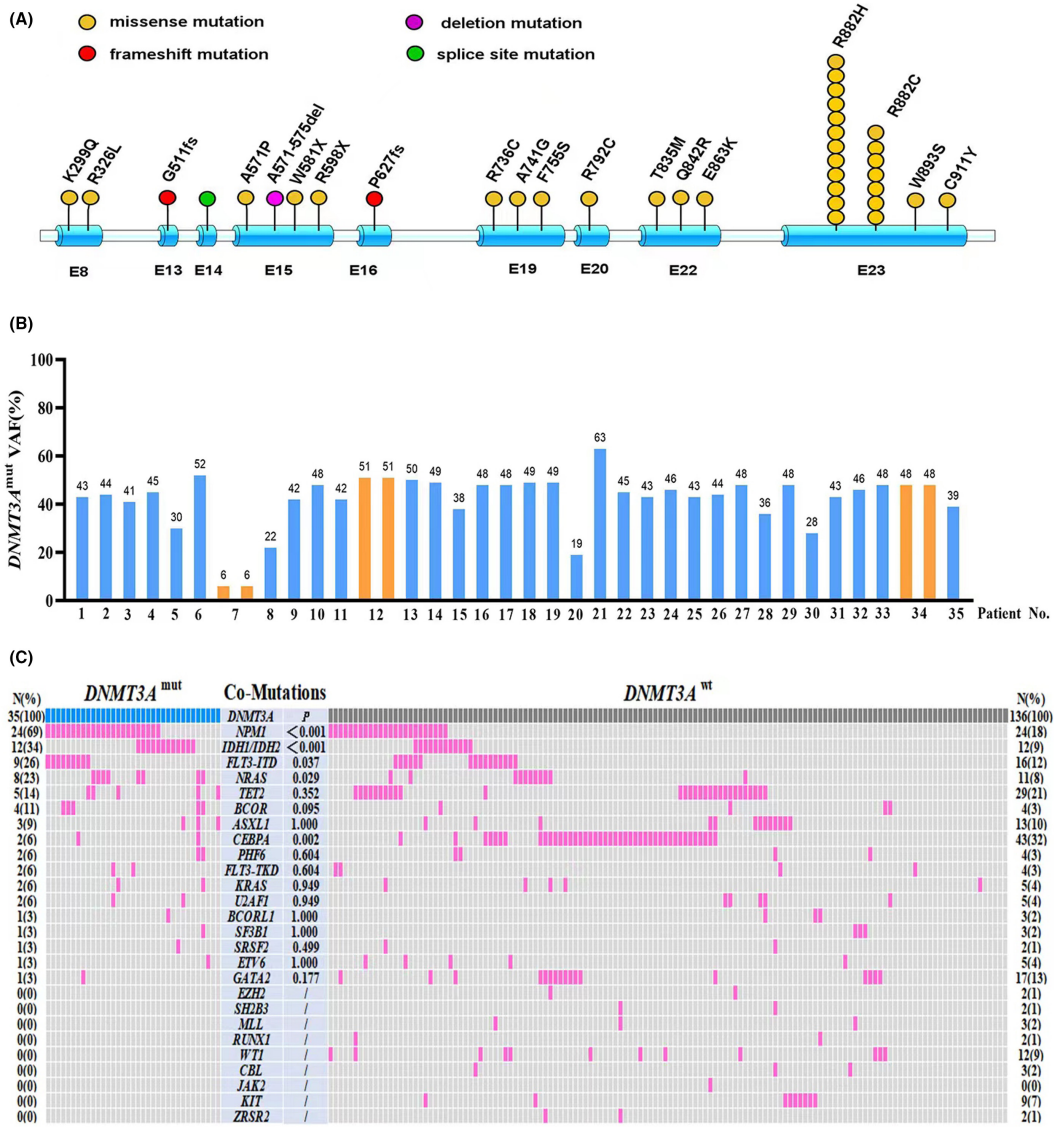
*DNMT3A* mutations and associated co‐mutations in 171 adult patients with de novo CN‐AML. (A) Structural diagram showing the location of mutations in *DNMT3A*. Each patient with *DNMT3A* mutation is designated with a circle. The color of the circle indicates the different types of mutations. (B) *DNMT3A* VAFs of all patient in this cohort. Blue bar indicates that the patient has single *DNMT3A* mutation. Orange bar indicates that the patient has two *DNMT3A* mutations. (C) Comparison of associated comutations and their frequency between *DNMT3A* ^mut^ and *DNMT3A* ^wt^ patients.

*DNMT3A* mutations were identified in 35 of 171 (20%) patients with de novo CN‐AML. Thirty‐two patients carried single mutations in the *DNMT3A* gene, and three patients harbored two *DNMT3A* variants (double mutations). *R882* missense mutations were more common variants (Figure 1A). Other *non‐R882* variants were detected in one patient respectively, including *W893S*,*C911Y*, *E863K*,*Q842R*,*T835M*, *R792C*,*F755S*,*A741G*,*R736C*,*A571P*,*R326L*,*K299Q*,*P627fs*,*G511fs*,*R598X*,*W581X*, *A571‐575del* (Figure 1A).

All 35 patients with *DNMT3A* ^mut^ harbored at least one or more companion comutations, with an average total of three mutations per patient (range:1–6), which was higher than *DNMT3A* ^wt^ patients with (mean:2; range: 1–6) (*p* < 0.001). A detailed mutations profiling is provided in Figure 1B,C. Notably, compared to those with *DNMT3A* ^wt^, patients with *DNMT3A* ^mut^ more frequently harbored *NPM1* (69% vs. 18%; *p* < 0.001), *IDH1/IDH2* (34% vs. 9%; *p* < 0.001), *FLT3‐ITD* (26% vs. 12%; *p* = 0.037), *NRAS* (29% vs. 8%; *p* = 0.029) mutations. In a similar fashion, patients with *DNMT3A* ^mut^ less frequently had *CEBPA* mutations (6% vs. 32%; *p* = 0.002). In this cohort, the median VAF value of *DNMT3A* mutations in 35 patients was 45% (6%–63%). Additionally, we observed *DNMT3A* mutations were presented as an ancestral mutation with higher or similar VAFs compared with other co‐mutations in 35 patients (data not shown). Based on data from comutations and their VAFs, we further identified three comutated patterns with high frequency in 35 patients, including *DNMT3A* ^mut^ *IDH1/IDH2* ^mut^ (*N* = 12, 34%), *DNMT3A* ^mut^ *NPM1* ^mut^ *FLT3‐ITD* ^mut^ (*N* = 9, 26%), and *DNMT3A* ^mut^ *NRAS* ^mut^ (*N* = 8, 23%). Among these three comutations, *DNMT3A* ^mut^ *IDH1/IDH2* ^mut^ and *DNMT3A* ^mut^ *NPM1* ^mut^ *FLT3‐ITD* ^mut^ existed independently of each other in *DNMT3A* ^mut^ patients of this cohort, whereas *DNMT3A* ^mut^ *NRAS* ^mut^ and *DNMT3A* ^mut^ *IDH1/IDH2* ^mut^ had overlaps in two patients, but was mutually exclusive with *DNMT3A* ^mut^ *NPM1* ^mut^ *FLT3‐ITD* ^mut^. Furthermore, we also observed that the ratios of *FLT3‐ITD* were lower than 0.5 in all of nine patients with *DNMT3A* ^mut^ *NPM1* ^mut^ *FLT3‐ITD* ^mut^. In addition, there were no significant differences in the VAFs of *DNMT3A* mutations between patients with or without *NPM1*(44.5% vs. 45%; *p* = 0.320), *FLT3‐ITD* (43% vs. 46.5%; *p* = 0.503), or *IDH1/IDH2* (46.5% vs. 44%; *p* = 0.469).

### 3.2 Clinical impact of DNMT3A VAF

We used the optimal cut‐off method to set the VAF of 42% as the cut‐off value, and further divided *DNMT3A* ^mut^ patients into two subgroups: *DNMT3A* ^High^ patients (*DNMT3A* VAF > 42%) and *DNMT3A* ^Low^ patients (*DNMT3A* VAF ≤42%). To classify double *DNMT3A* mutant cases into the *DNMT3A* ^High^ or *DNMT3A* ^Low^ group, the higher VAF of the two mutations was used.

**FIGURE 2:**
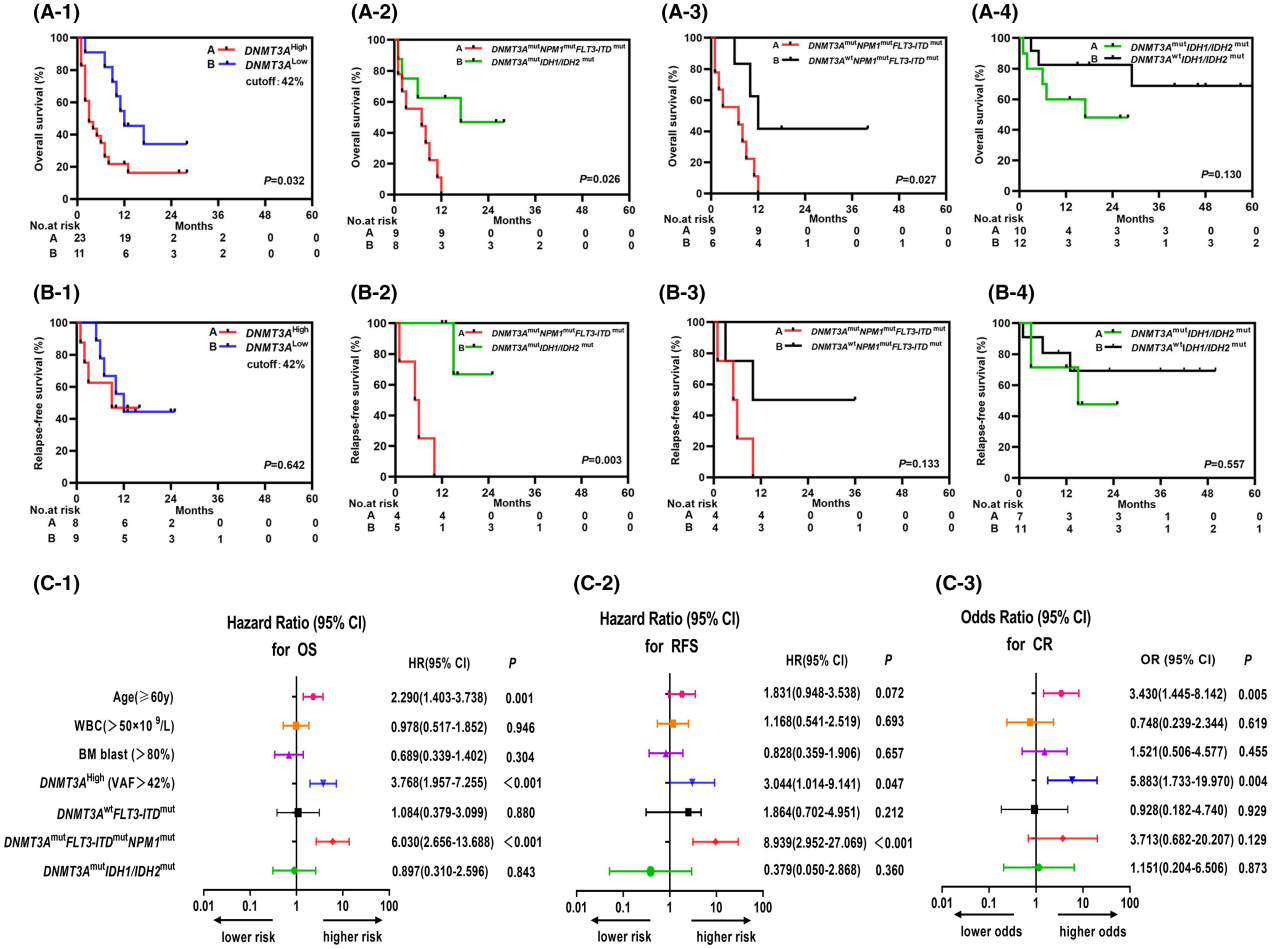
Prognosis effects of *DNMT3A* ^mut^ VAF and two comutations on adult patients with de novo CN‐AML. Kaplan–Meier survival curves for OS and RFS in *DNMT3A* ^High^ versus *DNMT3A* ^Low^ (A‐1,B‐1); as well as in *DNMT3A* ^mut^ *NPM*1^mut^ *FLT3‐ ITD* ^mut^ and *DNMT3A* ^mut^ *IDH1/IDH2* ^mut^ (A‐2,B‐2), in *DNMT3A* ^mut^ *NPM*1^mut^ *FLT3‐ ITD* ^mut^ and *DNMT3A* ^wt^ *NPM*1^mut^ *FLT3‐ITD* ^mut^ (A‐3,B‐3), in *DNMT3A* ^mut^ *IDH1/IDH2* ^mut^ and *DNMT3A* ^wt^ *IDH1/IDH2* ^mut^ (A‐4,B‐4). Cox‐proportional hazard regression models were analyzed for OS(C‐1) and RFS(C‐2). Logistic regression models were analyzed for CR(C‐3).

Biological features and patient outcomes were compared between these two subgroups (Table 1). To exclude the clinical effects of *DNMT3A* mutation types and comutations, here we evaluated these mutational features between the both subgroups. Our data showed that the number of patients with *R882* or *non‐R882*, comutated genes in the *DNMT3A* ^High^ subgroup were similarly distributed as those in *DNMT3A* ^Low^ subgroup. For biological features at diagnosis, patients with *DNMT3A* ^High^ had significantly increased white blood cell (WBC) counts (median:50.915 vs. 2.33 × 10^9^/L; *p <* 0.001) and a higher lactate dehydrogenase (LDH) level (median:582.85 vs. 333.5 U/mL; *p* = 0.027) than those with *DNMT3A* ^Low^. Other biological features (including age, sex, Hb levels, platelet counts, BM blast percentages) were not significantly different between *DNMT3A* ^High^ and *DNMT3A* ^Low^ subgroups. In this cohort, 32 patients further received therapy, whereas three patients did not receive any therapy. *DNMT3A* ^High^ subgroup had a greater number of patients receiving high‐intensity induction than *DNMT3A* ^Low^ subgroup, whereas most of patients with *DNMT3A* ^Low^receiving low‐intensity induction due to associated hypoproliferative AML. In addition, none of patients received allo‐HSCT in the entire *DNMT3A* ^mut^ group. Among patients receiving induction therapy, patients with *DNMT3A* ^High^ showed a significantly lower CR rate (38% vs. 82%; *p* = 0.015). In terms of survival, patients with *DNMT3A* ^High^ had a shorter OS (median: 3 vs. 12 months; *p* = 0.032) than those with *DNMT3A* ^Low^ (Figure 2 A1), but no statistical difference on RFS (median: 6 vs. 12 months; *p* = 0.642) was detected between them (Figure 2 B1). In multivariable analyses, *DNMT3A* ^High^ had independent effects on worse OS (hazard ratio [HR] = 3.768, 95% CI, 1.957–7.255; *p <* 0.001) (Figure 2 C1), and lower CR rate (odds ratio [OR] = 5.883, 95% CI, 1.733–19.970; *p* = 0.004) (Figure 2 C3).

**TABLE 1:** Comparison of clinical impact between *DNMT3A* ^High^ and *DNMT3A* ^Low^.

Characteristics	Total cohort (*N* = 35)	DNMT3A^High^ (VAF>42%) (*N* = 24)	DNMT3A^Low^ (VAF≤42%) (*N* = 11)	*p* value
Age				
≥60	16 (46)	13 (54)	3 (27)	0.138
<60	19 (54)	11 (46)	8 (73)	
Sex				
Male	16 (46)	11 (46)	5 (45)	0.983
Female	19 (54)	13 (54)	6 (55)	
Laboratory data median (range)				
Hb (g/L)	80.0 (46.0–139.0)	81.0 (46.00–138.00)	78.0 (46.0–139.0)	0.657
WBC (×10^9^/L)	24.6 (0.75–283.79)	50.915 (0.75–283.79)	2.33 (0.98–13.30)	**<0.001**
PLT (×10^9^/L)	76.0 (8.0–298.0)	76.5 (9.0–298.0)	70.0 (8.0–153.0)	0.831
BM blast (%)	49.0 (20.0–94.0)	57.5 (20.0–94.0)	40.0 (22.0–86.0)	0.095
LDH (U/mL)	427.0 (140.0–2210.0)	582.85 (140.0–2210.0)	333.5 (159.0–620.0)	**0.027**
Mutation type, *N* (%)				
R882	19 (54)	13 (54)	6 (55)	0.983
Non‐R882	16 (46)	11 (46)	5 (45)	
Co‐mutation				
NPM1	24 (69)	18 (75)	6 (55)	0.413
IDH	12 (34)	8 (33)	4 (36)	1.00
FLT3‐ITD	9 (26)	5 (21)	4 (36)	0.576
NRAS	8 (23)	7 (29)	1 (9)	0.379
TET2	5 (14)	4 (17)	1 (9)	1.00
BCOR	4 (11)	1 (4)	3 (27)	0.082
CEBPA	2 (6)	1 (4)	1 (9)	0.536
PHF6	2 (6)	1 (4)	1 (9)	0.536
Treatment, *N* (%)				
High intensity	18 (51)	15 (63)	3 (27)	**0.044**
Low intensity	14 (40)	6 (25)	8 (73)	
Outcome				
CR, *N* (%)	18 (51)	9 (38)	9 (82)	**0.015**
Median OS (range), months	7 (1–28)	3 (1–28)	12 (2–28)	**0.032**
Median RFS (range), months	10 (1–25)	6 (1–16)	12 (5–25)	**0.642**

*Note*: The bold values indicate *p* value < 0.05.

### 3.3 Clinical impact of two different DNMT3A ^mut^ comutations

To address the clinical impact of *DNMT3A* mutations in more detail and because of the high prevalence and mutual exclusion of two comutations of *DNMT3A* ^mut^, we next analyzed clinical features and outcomes of these two comutations, including *DNMT3A* ^mut^ *NPM1* ^mut^ *FLT3‐ITD* ^mut^ and *DNMT3A* ^mut^ *IDH1/IDH2* ^mut^.

We first compared two subgroups of patients with these two different comutated‐genetypes each other (Table 2). Similarly, we also compared *DNMT3A* ^mut^ VAF and type (*R882* and *non‐R882*) between both subgroups, and no significant statistical differences were observed. Our data further showed that these two subgroups were similar for biological features at diagnosis, including age, sex, Hb levels, WBC counts, platelet counts, BM blast percentages, and LDH levels. Regarding treatment types, there were no differences for the number of patients who received different induction therapies between the two subgroups. We observed that patients with *DNMT3A* ^mut^ *NPM1* ^mut^ *FLT3‐ITD* ^mut^ were associated with poorer OS (median:7 vs. 15 months; *p* = 0.026) (Figure 2 A2) and RFS (median: 5.5 vs. 15 months; *p* = 0.003) (Figure 2 B2) compared to those with *DNMT3A* ^mut^ *IDH1/IDH2* ^mut^, though there was no significant difference on CR rate between these two subgroups (Table 2).

**TABLE 2:** Comparison of clinical impact between the two *DNMT3A* ^mut^ comutaions.

Characteristics	*DNMT3A* ^mut^ *NPM*1^mut^ *FLT3‐ITD* ^mut^ (*N* = 9)	*DNMT3A* ^mut^ *IDH1/IDH*2^mut^ (*N* = 8)	*p* value
Age, *N* (%)			
≥60	2 (22)	4 (50)	0.335
<60	7 (78)	4 (50)	
Sex, *N* (%)			
Male	5 (56)	4 (50)	1.00
Female	4 (44)	4 (50)	
Laboratory data, Median (range)			
Hb (g/L)	78.5 (52.0–103.0)	77.0 (53.0–139.0)	1.00
WBC (×10^9^/L)	21.7 (0.98–283.79)	7.815 (1.25–123.71)	0.386
PLT (×10^9^/L)	52.0 (33.0–143.0)	85.0 (61.0–298.0)	0.124
BM blast (%)	49.0 (22.0–88.0)	42.9 (20.0–86.0)	0.630
LDH (U/mL)	339.0 (195.0–1981.0)	391.0 (157.0–866.0)	0.749
DNMT3A VAF			
High	5 (56)	4 (50)	1.00
Low	4 (44)	4 (50)	
Mutation type, *N* (%)			
R882	6 (67)	4 (50)	0.637
Non‐R882	3 (33)	4 (50)	
Treatment, *N* (%)			
High intensity	4 (44)	5 (63)	0.637
Low intensity	5 (56)	3 (38)	
Outcome			
CR, *N* (%)	4 (44)	5 (63)	0.637
Median OS (range), mo	7 (1–12)	15 (1–28)	**0.026**
Median RFS (range), mo	5.5 (1–10)	15 (12–25)	**0.003**

To investigate the interaction impact of *DNMT3A* mutations and comutations, we also compared biological and clinical outcomes of patients harboring *NPM1* ^mut^ *FLT3‐ ITD* ^mut^ (*FLT3‐ITD* ratio <0.5) genetype when with or without *DNMT3A* mutations, and patients harboring *IDH1/IDH2* ^mut^ when with or without *DNMT3A* mutations (Table 3). When comparing *DNMT3A* ^mut^ *NPM1* ^mut^ *FLT3‐ITD* ^mut^ and *DNMT3A* ^wt^ *NPM1* ^mut^ *FLT3‐ITD* ^mut^ subgroups, most of the features were similar, including age, sex, Hb levels, WBC counts, platelet counts, BM blast percentages, and LDH levels, treatment, CR rate, RFS, only for OS, patients with *DNMT3A* ^mut^ *NPM1* ^mut^ *FLT3‐ITD* ^mut^ revealed a shorter OS (median: 7 vs. 11 months; *p* = 0.027) than those with *DNMT3A* ^wt^ *NPM1* ^mut^ *FLT3‐ITD* ^mut^ (Figure 2 A3). When comparing *DNMT3A* ^mut^ *IDH1/IDH2* ^mut^ and *DNMT3A* ^wt^ *IDH1/IDH2* ^mut^ subgroups, patients with *DNMT3A* ^mut^ *IDH1/IDH2* ^mut^ presented with higher platelet counts (median: 85.0 vs. 37.0 × 10^9^/L; *p* = 0.009) and a lower BM blast percentages (median: 42.9 vs. 81.5%; *p* = 0.040) than those with *DNMT3A* ^wt^ *IDH1/IDH2* ^mut^, but no significant differences were detected for other biological features and their impacts on clinical outcomes. In multivariable analyses, *DNMT3A* ^mut^ *NPM1* ^mut^ *FLT3‐ITD* ^mut^ independently affected worse OS with a HR for the risk of death of 6.030 (95% CI, 2.656–13.688; *p <* 0.001) (Figure 2 C1), and worse RFS with an HR of 8.939 (95% CI, 2.952–27.069; *p <* 0.001) (Figure 2 C2). In contrast, *DNMT3A* ^mut^ *IDH1/IDH2* ^mut^ was not an independent factor for impacting CR rates, OS, and RFS (Figure 2 C1–3).

**TABLE 3:** Comparison of clinical impact between two comutaions with or without *DNMT3A* ^mut^.

Characteristics	*DNMT3A* ^mut^ *NPM1* ^mut^ *FLT3‐ITD* ^mut^ (*N* = 9)	*DNMT3A* ^wt^ *NPM1* ^mut^ *FLT3‐ITD* ^mut^ (*N* = 6)	*p* value	*DNMT3A* ^mut^ *IDH1/IDH2* ^mut^ (*N* = 8)	*DNMT3A* ^wt^ *IDH1/IDH2* ^mut^ (*N* = 8)	*p* value
Age, *N* (%)						
≥60	2 (22)	1 (17)	1.00	4 (50)	1 (13)	0.282
<60	7 (78)	5 (83)		4 (50)	7 (87)	
Sex, *N* (%)						
Male	5 (56)	1 (17)	0.287	4 (50)	3 (38)	1.00
Female	4 (44)	5 (83)		4 (50)	5 (63)	
Laboratory data, Median (range)						
Hb (g/L)	78.0 (52.0–103.0)	75.0 (72.0–92.0)	0.841	77.0 (53.0–139.0)	73.5 (45.0–117.0)	0.494
WBC (×10^9^/L)	21.7 (0.98–283.79)	49.53 (2.20–133.17)	0.593	7.815 (1.25–123.71)	3.305 (2.05–95.26)	0.401
PLT (×10^9^/L)	52.0 (33.0–143.0)	40.0 (22.0–108.0)	0.096	85.0 (61.0–298.0)	37.0 (13.0–113.0)	**0.009**
BM blast (%)	49.0 (22.0–88.0)	83.0 (50.0–88.0)	0.181	42.9 (20.0–86.0)	81.5 (20.0–91.0)	**0.040**
LDH (U/mL)	339.0 (195.0–1981.0)	316.0 (258.3–1540.0)	0.808	391.0 (157.0–866.0)	364.5 (166.0–634.8)	0.817
Treatment, *N* (%)						
High intensity	4 (44)	5 (83)	0.287	5 (63)	5 (63)	1.00
Low intensity	5 (56)	1 (17)		3 (38)	3 (38)	
Outcome						
CR, *N* (%)	4 (44)	4 (67)	0.608	5 (63)	7 (88)	0.569
Median OS (range), months	7 (1–12)	11 (6–40)	**0.027**	15 (1–28)	16.5 (3–48)	0.130
Median RFS (range), months	5.5 (1–10)	10 (3–36)	0.133	15 (12–25)	14 (1–46)	0.557

*Note*: The bold values indicate *p* value < 0.05.

## 4 DISCUSSION

In this study, we further assessed the genetic characteristics of *DNMT3A* mutations in adult patients with de novo CN‐AML using targeted NGS with a panel of 34 genes associated with myeloid leukemia. In accordance with previous studies,^9^, ^18^, ^19^, ^26^, ^27^, ^28^, ^29^, ^30^, ^31^, ^32^, ^33^, ^34^, ^35^, ^36^ we detected *DNMT3A* mutations with a frequency of 20% in adult patients with primary CN‐AML, and most of the mutations clustered at the *R882* site in exon 23. Moreover, all *DNMT3A* ^mut^ patients of this cohort harbored one or more additional mutations, of which the majority of patients had a relatively higher or similar VAF compared with other comutated genes, strengthening previous data that reported *DNMT3A* ^mut^ were presented as ancestral clone or preleukemia clone in AML.^12^, ^37^ Previous findings reported that *DNMT3A* mutations had a significant association with *NPM1* and *IDH1/IDH2* mutations, of which ~60% of *DNMT3A* ^mut^ cases having *NPM1*, and more often (~30%) displaying a significant co‐mutation pattern with *NPM1* and *FLT3‐ITD* mutations, and had a mutually exclusive relationship with *CEBPA* mutations in AML patients.^18^, ^19^, ^20^ Similarly, our data still showed that *DNMT3A* mutations had a significant association with *NPM1*, *FLT3‐ITD*, and *IDH1/IDH2* mutations, and but an inverse correlation with *CEBPA* mutations in CN‐AML patients. In addition, we found that *DNMT3A* mutations had a positive association with *NRAS* mutations in our patients. Furthermore, by an analysis of comutations, we identified two critical comutated genetypes with a high frequency, including *DNMT3A* ^mut^ *IDH1/IDH2* ^mut^ and *DNMT3A* ^mut^ *NPM1* ^mut^ *FLT3‐ITD* ^mut^. Although *DNMT3A* ^mut^ *IDH1/IDH2* ^mut^ and *DNMT3A* ^mut^ *NPM1* ^mut^ *FLT3‐ITD* ^mut^ comutations had been reported in other studies, their mutation features were rarely described. Here we found that *DNMT3A* ^mut^ *IDH1/IDH2* ^mut^ and *DNMT3A* ^mut^ *NPM1* ^mut^ *FLT3‐ITD* ^mut^ were mutually exclusive in *DNMT3A* ^mut^ patients, and that the mutation ratios of *FLT3‐ITD* were lower than 0.5 in all of the patients with *DNMT3A* ^mut^ *NPM1* ^mut^ *FLT3‐ITD* ^mut^. The above data strongly indicated that *DNMT3A* ^mut^ *NPM1* ^mut^ *FLT3‐ITD* ^mut^ and *DNMT3A* ^mut^ *IDH1/IDH2* ^mut^ might be two different genetic subgroups of *DNMT3A* ^mut^ patients with CN‐AML.

We first investigated the biological and clinical impact of *DNMT3A* ^mut^ VAF on CN‐AML, which be stratified by the cut‐off value of 42%. For biological features, we observed that *DNMT3A* ^High^ patients at diagnosis had a significantly higher WBC counts and a trend for higher BM blast percentage compared with *DNMT3A* ^Low^ ones, further strengthing a previous report by Narayanan et al.^38^ that that *DNMT3A* ^High^ (≥44%) patients presented with leukocytosis and higher blast counts, and they further demonstrated that *DNMT3A* VAF had a positive correlations with WBC counts in AML patients. Combining these two data sets suggested that higher WBC counts and BM blast percentage might be unique biological features for patients with CN‐AML with *DNMT3A* ^High^. In addition, we also observed another striking feature with more elevated serum LDH levels in *DNMT3A* ^High^ cases in our cohort. As far as we know, this is the first description of such an association in *DNMT3A* ^mut^ AML patients. With regard to clinical outcomes, in univariable and multivariable analyses, we found that *DNMT3A* ^High^ conferred an unfavorable effect on CR rate and OS, but did not show its negative impact on RFS in these patients. Although the predictive results of *DNMT3A* VAF are rarely reported, our findings were similar to previous reports. An analysis of 104 patients with *DNMT3A*‐mutated AML led by *Narayanan* et al.^38^ showed that high *DNMT3A* VAF was associated with more inferior OS and EFS, but had no impact on CR rate in univariable analyses, but in multivariable analyses, the adverse effect of *DNMT3A* ^High^ only on OS but not on EFS in the CN‐AML subset. *Linch* et al.^39^ reported that high *DNMT3A R882* ^mut^ VAF (≥47%) also presented worse effects on CR rate and OS in univariable analyses, but were not significant in multivariable analyses. Yuan et al.^40^ reported that higher *DNMT3A R882* ^mut^ VAF (≥39%) had a shorter OS than those with a lower *DNMT3A R882* ^mut^ VAF.

Based on the comutations features of *DNMT3A* ^mut^, we further investigated the clinical effects of two comutations, including *DNMT3A* ^mut^ *NPM1* ^mut^ *FLT3‐ITD* ^mut^ and *DNMT3A* ^mut^ *IDH1/IDH2* ^mut^, which were considered as the two genetic subgroups of *DNMT3A* ^mut^ patients with de novo CN‐AML in this study. We observed that patients with *DNMT3A* ^mut^ *NPM1* ^mut^ *FLT3‐ITD* ^mut^ had significantly worse OS and RFS than those with *DNMT3A* ^mut^ *IDH1/IDH2* ^mut^. To better understand the effect of *DNMT3A* mutations in CN‐AML, we compared the clinical impact of *NPM1* ^mut^ *FLT3‐ ITD* ^mut^ and *IDH1/IDH2* ^mut^ when with or without the genetic context of *DNMT3A* mutations. Recently, the 2022 ELN guideline has updated *FLT3‐ITD* as an intermediate risk marker irrespective of *NPM1* mutational status.^41^Our data showed that *DNMT3A* mutations had a significantly poorer effect on OS on patients with *NPM1* ^mut^ *FLT3‐ITD* ^mut^ (*FLT3‐ITD‐*ratio <0.5) genetype, further suggesting that *DNMT3A* mutations could reduce favorable prognosis effect of *NPM1* ^mut^ *FLT3‐ITD* ^mut^ (*FLT3‐ITD‐*ratio <0.5) genetype. In contrast, we did not observe significant differences on clinical outcomes between *DNMT3A* ^mut^ *IDH1/IDH2* ^mut^ and *DNMT3A* ^wt^ *IDH1/IDH2* ^mut^ subgroups, indicating that *DNMT3A* mutations could not change the clinical prognosis of *IDH1/IDH2* mutations. In multivariable analyses, the *DNMT3A* ^mut^ *NPM1* ^mut^ *FLT3‐ITD* ^mut^ (*FLT3‐ITD‐*ratio <0.5) was still independently associated with worse OS and RFS in this cohort. The above data strongly indicated that *DNMT3A* mutations could generate different prognostic effects when combined with different comutations. In addition, although *DNMT3A* ^mut^ *IDH1/IDH2* ^mut^ had no significant prognosis impact, we found that patients with this mutated genetype presented biological features such as higher platelet counts and a lower BM blast percentage in comparison to those with *DNMT3A* ^wt^ *IDH1/IDH2* ^mut^. To the best of our knowledge, the clinical impact of the *DNMT3A* ^mut^ *IDH1/IDH2* ^mut^ was barely reported, whereas the clinical implications of the *DNMT3A* ^mut^ *NPM1* ^mut^ *FLT3‐ITD* ^mut^ were described in only a few reports. A report led by Loghavi et al.^42^ showed that *DNMT3A* ^mut^ *NPM1* ^mut^ *FLT3‐ITD* ^mut^ had a shorter effect on EFS and a trend for shorter OS among AML old patients in comparison to those within other mutation subgroups. Another recent study cohort conducted by Bezerra et al.^43^ reported that the *DNMT3A* ^mut^ *NPM1* ^mut^ *FLT3‐ITD* ^mut^ had worse effects on OS and DFS, which was similar to our findings.

## 5 CONCLUSIONS

In summary, this study more detailly refined the biological and clinical prognostic effects of *DNMT3A* ^mut^ in adult patients with de novo CN‐AML. Our findings highlighted that *DNMT3A* ^mut^ VAF and its two comutations had their specific clinical consequences. We found that high *DNMT3A* VAF was associated with higher WBC counts and BM blast percentage than low *DNMT3A* VAF, and had an independent effect on lower CR rate and shorter OS. We also identified that *DNMT3A* ^mut^ *NPM1* ^mut^ *FLT3‐ITD* ^mut^ exerted an independent worse impact on OS and RFS. In contrast, the patients with *DNMT3A* ^mut^ *IDH1/IDH2* ^mut^ had relatively favorable prognoses, but manifested as higher platelet counts and a lower BM blast percentage in CN‐AML patients than those with *DNMT3A* ^wt^ *IDH1/IDH2* ^mut^. However, there were several limitations to the current study because of a small sample size and its retrospective nature. Our findings need to be validated in a more extensive and prospective cohort, which might be the potentially helpful in predicting clinical outcomes.

## AUTHOR CONTRIBUTIONS

**Xian Chen:** Data curation (equal); formal analysis (equal); investigation (equal); methodology (equal); writing – original draft (equal). **Chuchu Tian:** Data curation (equal); formal analysis (equal); investigation (equal); methodology (equal). **Zhuanghui Hao:** Data curation (equal); formal analysis (equal); investigation (equal); methodology (equal); writing – original draft (equal). **Lingang Pan:** Data curation (equal); formal analysis (equal); investigation (equal); methodology (equal); writing – original draft (equal). **Minglin Hong:** Data curation (equal); formal analysis (equal); investigation (equal); methodology (equal). **Wei Wei:** Data curation (equal); formal analysis (equal); investigation (equal); methodology (equal). **Daniel Muteb Muyey:** Data curation (equal); formal analysis (equal); investigation (equal); methodology (equal). **Hongwei Wang:** Conceptualization (equal); funding acquisition (equal); project administration (equal); writing – review and editing (equal). **Xiuhua Chen:** Conceptualization (equal); data curation (equal); formal analysis (equal); funding acquisition (equal); investigation (equal); methodology (equal); project administration (equal); writing – review and editing (equal).

## FUNDING INFORMATION

This work was supported by the National Natural Science Foundation of China (nos 81500104; 81670126), The Shanxi Natural Science Foundation of China (nos 201801D221409; 201801D111003), Graduate Innovation Fund of Shanxi Province.

## CONFLICT OF INTEREST STATEMENT

All the authors declare they have no competing interests.

## References

[1] Okano M , Xie S , Li E . Cloning and characterization of a family of novel mammalian DNA (cytosine‐5) methyltransferases. Nat Genet. 1998;19:219‐220. 9662389 10.1038/890

[2] Okano M , Bell DW , Haber DA , Li E . DNA methyltransferases *Dnmt3a* and *Dnmt3b* are essential for de novo methylation and mammalian development. Cell. 1999;99:247‐257. 10555141 10.1016/s0092-8674(00)81656-6

[3] Dhawan S , Tschen SI , Zeng C , et al. DNA methylation directs functional maturation of pancreatic beta cells. J Clin Invest. 2015;125:2851‐2860. 26098213 10.1172/JCI79956 PMC4563682

[4] Liao J , Karnik R , Gu H , et al. Targeted disruption of *DNMT1*, *DNMT3A* and *DNMT3B* in human embryonic stem cells. Nat Genet. 2015;47:469‐478. 25822089 10.1038/ng.3258 PMC4414868

[5] Challen GA , Sun D , Mayle A , et al. Dnmt3a and Dnmt3b have overlapping and distinct functions in hematopoietic stem cells. Cell Stem Cell. 2014;15:350‐364. 25130491 10.1016/j.stem.2014.06.018 PMC4163922

[6] Challen GA , Sun D , Jeong M , et al. Dnmt3a is essential for hematopoietic stem cell differentiation. Nat Genet. 2012;44:23‐31. 10.1038/ng.1009 PMC3637952 22138693

[7] Hu N , Strobl‐Mazzulla P , Sauka‐Spengler T , Bronner ME . DNA methyltransferase3A as a molecular switch mediating the neural tube‐to‐neural crest fate transition. Genes Dev. 2012;26:2380‐2385. 23124063 10.1101/gad.198747.112 PMC3489996

[8] Wu Z , Huang K , Yu J , et al. Dnmt3a regulates both proliferation and differentiation of mouse neural stem cells. J Neurosci Res. 2012;90:1883‐1891. 22714992 10.1002/jnr.23077 PMC3418436

[9] Ley TJ , Ding L , Walter MJ , et al. *DNMT3A* mutations in acute myeloid leukemia. N Engl J Med. 2010;363:2424‐2433. 21067377 10.1056/NEJMoa1005143 PMC3201818

[10] Yan XJ , Xu J , Gu ZH , et al. Exome sequencing identifies somatic mutations of DNA methyltransferase gene DNMT3A in acute monocytic leukemia. Nat Genet. 2011;43(4):309‐315. 21399634 10.1038/ng.788

[11] Roller A , Grossmann V , Bacher U , et al. Landmark analysis of *DNMT3A* mutations in hematological malignancies. Leukemia. 2013;27:1573‐1578. 23519389 10.1038/leu.2013.65

[12] Shlush LI , Zandi S , Mitchell A , et al. Identification of pre‐leukaemic haematopoietic stem cells in acute leukaemia. Nature. 2014;506:328‐333. 24522528 10.1038/nature13038 PMC4991939

[13] Klco JM , Miller CA , Griffith M , et al. Association between mutation clearance after induction therapy and outcomes in acute myeloid leukemia. JAMA. 2015;314:811‐822. 26305651 10.1001/jama.2015.9643 PMC4621257

[14] Ivey A , Hills RK , Simpson MA , et al. Assessment of minimal residual disease in standard‐risk AML. N Engl J Med. 2016;374:422‐433. 26789727 10.1056/NEJMoa1507471

[15] Ho PA , Kutny MA , Alonzo TA , et al. Leukemic mutations in the methylation‐associated genes *DNMT3A* and *IDH2* are rare events in pediatric AML: a report from the Children's oncology group. Pediatr Blood Cancer. 2011;57:204‐209. 21504050 10.1002/pbc.23179 PMC3115394

[16] Thol F , Heuser M , Damm F , Klusmann J‐HH , Reinhardt K , Reinhardt D . *DNMT3A* mutations are rare in childhood acute myeloid leukemia. Haematologica. 2011;96:1238‐1240. 21685466 10.3324/haematol.2011.046839 PMC3148921

[17] Shiba N , Taki T , Park MJJ , et al. *DNMT3A* mutations are rare in childhood acute myeloid leukaemia, myelodysplastic syndromes and juvenile myelomonocytic leukaemia. Br J Haematol. 2012;156:413‐414. 21981547 10.1111/j.1365-2141.2011.08879.x

[18] Gaidzik VI , Schlenk RF , Paschka P , et al. Clinical impact of *DNMT3A* mutations in younger adult patients with acute myeloid leukemia: results of the AML study group (AMLSG). Blood. 2013;121:4769‐4777. 23632886 10.1182/blood-2012-10-461624

[19] Cancer Genome Atlas Research N . Genomic and epigenomic landscapes of adult de novo acute myeloid leukemia. N Engl J Med. 2013;368:2059‐2074. 23634996 10.1056/NEJMoa1301689 PMC3767041

[20] Gale RE , Lamb K , Allen C , et al. Simpson's paradox and the impact of different *DNMT3A* mutations on outcome in younger adults with acute myeloid leukemia. J Clin Oncol. 2015;33:2072‐2083. 25964253 10.1200/JCO.2014.59.2022

[21] Holz‐Schietinger C , Matje DM , Harrison MF , Reich NO . Oligomerization of *DNMT3A* controls the mechanism of *de nov*o DNA methylation. J Biol Chem. 2011;286:41479‐41488. 21979949 10.1074/jbc.M111.284687 PMC3308859

[22] Qu Y , Lennartsson A , Gaidzik VI , et al. Differential methylation in CN‐AML preferentially targets non‐CGI regions and is dictated by *DNMT3A* mutational status and associated with predominant hypomethylation of HOX genes. Epigenetics. 2014;9:1108‐1119. 24866170 10.4161/epi.29315 PMC4164496

[23] Meyer SE , Qin T , Muench DE , et al. Dnmt3a haploinsufficiency transforms *Flt3*ITD myeloproliferative disease into a rapid, spontaneous, and fully penetrant acute myeloid leukemia. Cancer Discov. 2016;6:501‐515. 27016502 10.1158/2159-8290.CD-16-0008 PMC4861898

[24] Yang L , Rodriguez B , Mayle A , et al. DNMT3A loss drives enhancer hypomethylation in FLT3‐ITD‐associated leukemias. Cancer Cell. 2016;29:922‐934. 27300438 10.1016/j.ccell.2016.05.003 PMC4908977

[25] Koya J , Kataoka K , Sato T , et al. *DNMT3A* R882 mutants interact with polycomb proteins to block haematopoietic stem and leukaemic cell differentiation. Nat Commun. 2016;7:10924. 27010239 10.1038/ncomms10924 PMC4820786

[26] Shen Y , Zhu YM , Fan X , et al. Gene mutation patterns and their prognostic impact in a cohort of 1,185 patients with acute myeloid leukemia. Blood. 2011;18:5593‐5503. 10.1182/blood-2011-03-343988 21881046

[27] Thol F , Damm F , Lüdeking A , et al. Incidence and prognostic influence of *DNMT3A* mutations in acute myeloid leukemia. J Clin Oncol. 2011;29:2889‐2896. 21670448 10.1200/JCO.2011.35.4894

[28] Hou HA , Kuo YY , Liu CY , et al. *DNMT3A* mutations in acute myeloid leukemia: stability during disease evolution and clinical implications. Blood. 2012;119:559‐568. 22077061 10.1182/blood-2011-07-369934

[29] Marcucci G , Metzeler KH , Schwind S , et al. Age‐related prognostic impact of different types of *DNMT3A* mutations in adults with primary cytogenetically normal acute myeloid leukemia. J Clin Oncol. 2012;30:742‐750. 22291079 10.1200/JCO.2011.39.2092 PMC3295550

[30] Renneville A , Boissel N , Nibourel O , et al. Prognostic significance of DNA methyltransferase 3A mutations in cytogenetically normal acute myeloid leukemia: a study by the acute Leukemia French association. Leukemia. 2012;26:1247‐1254. 22289988 10.1038/leu.2011.382

[31] Ribeiro AF , Pratcorona M , Erpelinck‐Verschueren C , et al. Mutant *DNMT3A*: a new marker of poor prognosis in acute myeloid leukemia. Blood. 2012;119:5824‐5831. 22490330 10.1182/blood-2011-07-367961

[32] Hou HA , Lin CC , Chou WC , et al. Integration of cytogenetic and molecular alterations in risk stratification of 318 patients with *de novo* non‐M3 acute myeloid leukemia. Leukemia. 2014;28:50‐58. 23929217 10.1038/leu.2013.236

[33] Tie R , Zhang T , Fu H , et al. Association between *DNMT3A* mutations and prognosis of adults with de novo acute myeloid leukemia: a systematic review and meta‐analysis. PLoS ONE. 2014;9:e96653. 24936645 10.1371/journal.pone.0093353 PMC4061003

[34] Patel JP , Gönen M , Figueroa ME , et al. Prognostic relevance of integrated genetic profiling in acute myeloid leukemia. N Engl J Med. 2012;366:1079‐1089. 22417203 10.1056/NEJMoa1112304 PMC3545649

[35] Pløen GG , Nederby L , Guldberg P , et al. Persistence of *DNMT3A* mutations at long‐term remission in adult patients with AML. Br J Haematol. 2014;167:478‐486. 25371149 10.1111/bjh.13062

[36] Sun Y , Shen H , Xu T , et al. Persistent DNMT3A mutation burden in *DNMT3A* mutated adult cytogenetically normal acute myeloid leukemia patients in long‐term remission. Leuk Res. 2016;49:102‐107. 27626217 10.1016/j.leukres.2016.09.001

[37] Corces‐Zimmerman RM , Hong WJ , Weissman IL , Medeiros BC , Majeti R . Preleukemic mutations in human acute myeloid leukemia affect epigenetic regulators and persist in remission. Proc Natl Acad Sci. 2014;111:2548‐2553. 24550281 10.1073/pnas.1324297111 PMC3932921

[38] Narayanan D , Pozdnyakova O , Hasserjian RP , Patel SS , Weinberg OK . Effect ofDNMT3Avariant allele frequency and double mutation on clinicopathologic features of patients with de novo AML. Blood Adv. 2021;5(11):2539‐2549. 34100902 10.1182/bloodadvances.2021004250 PMC8238486

[39] Linch DC , Hills RK , Burnett AK , Gale RE . The clinical impact of mutant DNMT3A R882 variant allele frequency in acute myeloid leukaemia. Br J Haematol. 2020;189(3):e81‐e86. 32004382 10.1111/bjh.16486

[40] Yuan XQ , Chen P , Du YX , et al. Influence of DNMT3A R882 mutations on AML prognosis determined by the allele ratio in Chinese patients. J Transl Med. 2019;17(1):220. 31291961 10.1186/s12967-019-1959-3 PMC6621981

[41] Döhner H , Wei AH , Appelbaum FR , et al. Diagnosis and management of AML in adults: 2022 recommendations from an international expert panel on behalf of the EL. Blood. 2022;140(12):1345‐1377. 35797463 10.1182/blood.2022016867

[42] Loghavi S , Zuo Z , Ravandi F , et al. Clinical features of de novo acute myeloid leukemia with concurrent *DNMT3A*, *FLT3* and *NPM1* mutations. J Hematol Oncol. 2014;7:74. 25281355 10.1186/s13045-014-0074-4 PMC4197326

[43] Bezerra MF , Lima AS , Piqué‐Borràs MR , et al. Co‐occurrence of *DNMT3A*, *NPM1*, *FLT3* mutations identifies a subset of acute myeloid leukemia with adverse prognosis. Blood. 2020;135(11):870‐875. 31977039 10.1182/blood.2019003339

